# Supplementary material for: Generating variability from motor primitives during infant locomotor development
Source: eLife. 2023 Jul 31;12:e87463. doi: 10.7554/eLife.87463 (PMC10390046; doi:10.7554/eLife.87463)
Supplement: Supplementary file 1. — (a) Summary of p-values associated with Wilcoxon tests. p-Values are considered significant for p<0.05 (dark gray) and a trend is considered between 0.05 and 0.1 (light gray) E1: birth; E2: 3 months old; E3: walking onset. (b) Individual data regarding the dimensionality of the signals: VAF for a modeling of four modules and number of modules to reach the threshold VAF (Figure 3B). The goodness of fit is considered sufficient above 75%. Subjects are in the same order than displayed in Figure 3E. (c) Individual data of 20 adults from Hinnekens et al., 2020 were used to plot adult landmarks (Figures 3 and 5). Note that those data are displayed for illustration: even though we retrieved raw data to apply the same filtering and normalization as in the rest of the article, data are not directly comparable since adult cycles were defined as step cycles (with a stance phase and a swing phase), whereas infant/toddler data were cut off as flexion and extension cycles (with an extension phase and a flexion phase). [file elife-87463-supp1.docx]

Supplementary file 1

Supplementary File 1a

Summary of p-values associated with Wilcoxon tests.

P-values are considered significant for p<0.05 (dark grey) and a trend is considered between 0.05 and 0.1 (light grey)

E1: birth / E2: 3 months old / E3: walking onset

|  | | **Stepping** | | | **Kicking** | | |
| --- | --- | --- | --- | --- | --- | --- | --- |
|  |  | **E1 - E2** | **E2 - E3** | **E1 - E3** | **E1 - E2** | **E2 - E3** | **E1 - E3** |
| **Cycle duration** | | .010 | <.001 | .002 | .003 | .009 | <.001 |
| **Proportion of phases** | | .577 | .216 | .019 | .622 | .021 | .012 |
| **Variability of cycle duration** | | .083 | <.001 | .002 | .021 | <.001 | <.001 |
| **IEV** | | .005 | .003 | .004 | .519 | .001 | <.001 |
| **VAF (Goodness of fit)**  **for 4 modules** | | .019 | <.001 | .002 | .850 | <.001 | <.001 |
| **For each individual's number of module** | **IRV** | .001 | <.001 | .002 | .424 | <.001 | <.001 |
|  | **IRS** | .365 | .168 | .049 | .301 | .034 | .021 |
|  | **SMAI** | .054 | <.001 | .002 | .042 | <.001 | <.001 |
|  | **STAI** | .240 | <.001 | .002 | .569 | <.001 | <.001 |
| **For a fixed number of 4 modules (methodological verification)** | **IRV** | .005 | <.001 | .002 | .470 | <.001 | <.001 |
|  | **IRS** | .206 | .002 | .010 | .910 | .002 | .001 |
|  | **SMAI** | .014 | <.001 | .004 | .110 | <.001 | <.001 |
|  | **STAI** | .102 | <.001 | .002 | .970 | <.001 | <.001 |

Supplementary File 1b

Individual data regarding the dimensionality of the signals: VAF for a modeling of 4 modules and number of modules to reach the threshold VAF (**Figure 3B**).

The goodness of fit is considered sufficient above 75%.

Subjects are in the same order than displayed in Figure 3E.

| **Subject ID** | **VAF (Goodness of fit) for 4 modules** | | | | |
| --- | --- | --- | --- | --- | --- |
|  | **Stepping** | | **Walking** | **Kicking** | |
|  | **Birth** | **3 months** |  | **3 months** | **Birth** |
| 11 | / | / | 0.566 | 0.742 | 0.866 |
| 8 | 0.801 | 0.709 | 0.626 | 0.763 | 0.741 |
| 4 | / | 0.638 | 0.671 | 0.790 | 0.700 |
| 16 | 0.774 | 0.696 | 0.552 | 0.749 | 0.766 |
| 3 | 0.847 | 0.716 | 0.601 | 0.761 | / |
| 7 | / | 0.717 | 0.626 | 0.756 | / |
| 15 | 0.713 | 0.680 | 0.562 | / | 0.787 |
| 17 | 0.773 | 0.684 | / | 0.789 | 0.806 |
| 6 | 0.673 | 0.700 | 0.561 | / | 0.717 |
| 14 | / | 0.659 | 0.600 | 0.673 | 0.636 |
| 12 | / | 0.746 | 0.534 | 0.775 | 0.782 |
| 2 | 0.760 | 0.654 | 0.644 | 0.764 | / |
| 10 | 0.832 | / | 0.596 | / | 0.759 |
| 13 | 0.789 | 0.712 | 0.600 | 0.719 | 0.786 |
| 18 | 0.655 | 0.722 | / | 0.769 | 0.738 |
| 1 | 0.764 | 0.782 | 0.574 | 0.684 | 0.773 |
| 9 | 0.762 | 0.664 | 0.546 | 0.795 | 0.682 |
| 5 | / | / | / | 0.745 | 0.700 |
| **Mean** | **0.762** | **0.699** | **0.591** | **0.752** | **0.749** |
| **SD** | **0.058** | **0.037** | **0.039** | **0.036** | **0.057** |

Supplementary File 1c

Individual data of 20 adults from Hinnekens et al. (2020) were used to plot adult landmarks (**Figure 3** and **Figure 5**). Note that those data are displayed for illustration: even though we retrieved raw data to apply the same filtering and normalization as in the rest of the paper, data are not directly comparable since adult cycles were defined as step cycles (with a stance phase and a swing phase) whereas infant/toddler data were cut off as flexion and extension cycles (with an extension phase and a flexion phase). As for infant data, indexes were computed as mean scores for each individual across 5 random combinations of 5 steps (see methods, data processing), which explains that the number of modules reported here is not necessarily an integer.

| **Adult** | **IEV** | **VAF** | **Module number to reach VAF>75%** | **IRV** | **IRS** | **SMAI** | **STAI** |
| --- | --- | --- | --- | --- | --- | --- | --- |
| 1 | 0.056 | 0.622 | 6 | 2.850 | 0.625 | 0.773 | 0.418 |
| 2 | 0.056 | 0.699 | 5.2 | 3.209 | 0.594 | 0.796 | 0.450 |
| 3 | 0.060 | 0.669 | 5 | 4.132 | 0.629 | 0.759 | 0.402 |
| 4 | 0.054 | 0.778 | 4 | 4.885 | 0.590 | 0.766 | 0.436 |
| 5 | 0.053 | 0.787 | 4 | 4.672 | 0.602 | 0.750 | 0.461 |
| 6 | 0.054 | 0.708 | 5.2 | 2.875 | 0.618 | 0.781 | 0.444 |
| 7 | 0.057 | 0.702 | 5.2 | 3.904 | 0.613 | 0.756 | 0.451 |
| 8 | 0.059 | 0.750 | 4.6 | 4.049 | 0.598 | 0.722 | 0.458 |
| 9 | 0.060 | 0.699 | 5 | 4.792 | 0.601 | 0.710 | 0.426 |
| 10 | 0.049 | 0.703 | 5 | 1.693 | 0.687 | 0.786 | 0.495 |
| 11 | 0.047 | 0.659 | 5.4 | 2.615 | 0.581 | 0.798 | 0.492 |
| 12 | 0.049 | 0.754 | 4.2 | 3.849 | 0.612 | 0.790 | 0.483 |
| 13 | 0.056 | 0.717 | 5 | 3.566 | 0.598 | 0.779 | 0.481 |
| 14 | 0.054 | 0.738 | 4.8 | 3.380 | 0.644 | 0.768 | 0.478 |
| 15 | 0.046 | 0.720 | 5 | 2.651 | 0.648 | 0.800 | 0.463 |
| 16 | 0.051 | 0.738 | 5 | 3.547 | 0.622 | 0.767 | 0.420 |
| 17 | 0.047 | 0.715 | 5 | 2.842 | 0.645 | 0.776 | 0.458 |
| 18 | 0.063 | 0.808 | 3.8 | 5.458 | 0.596 | 0.715 | 0.421 |
| 19 | 0.049 | 0.654 | 5.8 | 2.752 | 0.597 | 0.788 | 0.428 |
| 20 | 0.055 | 0.723 | 5 | 3.655 | 0.647 | 0.741 | 0.430 |
| **Landmark (mean)** | **0.054** | **0.717** | **4.910** | **3.569** | **0.617** | **0.766** | **0.450** |
| **Standard deviation** | **0.005** | **0.046** | **0.564** | **0.929** | **0.026** | **0.027** | **0.027** |
